# Circadian Regulation of the Biology of Allergic Disease: Clock Disruption Can Promote Allergy

**DOI:** 10.3389/fimmu.2020.01237

**Published:** 2020-06-12

**Authors:** Atsuhito Nakao

**Affiliations:** ^1^Department of Immunology, Faculty of Medicine, University of Yamanashi, Kofu, Japan; ^2^Atopy Research Center, Juntendo University School of Medicine, Tokyo, Japan

**Keywords:** circadian clock, allergic disease, epithelial barrier function, immune response, clock disruption

## Abstract

Allergic diseases such as allergic rhinitis, asthma, atopic dermatitis, and food allergy are characterized by epithelial barrier dysfunction and deregulated immune responses. Components of the circadian clock interact with critical elements of epithelial barrier function and immune responses, and regulate the biological processes on a 24-h cycle at steady state. This may represent an anticipatory defense response to day–night fluctuation of attack by noxious stimuli such as pathogens in the environment. This review will summarize clock control of epithelial barrier function and immune responses associated with allergic disease and offer novel insights and opportunities into how clock dysfunction impacts allergic disease. Importantly, perturbation of normal clock activity by genetic and environmental disturbances, such as chronic light cycle perturbations or irregular eating habits, deregulates epithelial barrier function and immune responses. This implies that the circadian clock is strongly linked to the fundamental biology of allergic disease, and that clock disruption can precipitate allergic disease by altering the epithelial barrier and immune functions. Given that contemporary lifestyles often involve chronic circadian disruptions such as shift work, we propose that lifestyle or therapeutic interventions that align the endogenous circadian clock with environmental cycles should be a part of the efforts to prevent or treat allergic disease in modern society.

## Introduction

Allergic diseases such as allergic rhinitis, asthma, atopic dermatitis, and food allergy are serious public and medical concerns due to their high prevalence and the harm they cause, in terms of both patient quality of life and socioeconomics (1). However, we do not fully understand why allergic disease is so prevalent in modern society. Hence, we need to understand previously unrecognized aspects of the biology of allergic disease.

Allergic disease is characterized by epithelial barrier dysfunction and deregulated immune response (allergic immune response) (2–4). For instance, in atopic dermatitis (AD), several components of skin barrier function, such as filaggrin, tight junction, and the microbiome are compromised in terms of quantity and/or quality, leading to increased cutaneous permeability (5). Consequently, the skin permits allergen penetration and releases epithelial cytokines (e.g., IL-33), which trigger an allergic immune response. Decreased regulatory T-cell activity in AD may also promote allergic immune responses (6).

The circadian clock is the endogenous timing-keeping mechanisms, by which living organisms fit their physiology and behavior to daily alterations in the rhythmic environment created by the earth's rotation (7). Recent studies highlight that the circadian clock underpins epithelial barrier function and immune responses under physiological conditions (8–12). The components of the circadian clock interact with critical elements or pathways of epithelial barrier function and immune responses, thereby regulating these biological processes on a 24-h cycle. This may represent an anticipatory defense response to day–night fluctuation of attack by pathogens and non-pathogenic insults in the outer environment (11, 13). In this review, I will summarize clock control of epithelial barrier function and immune responses that are associated with allergic disease and provide insight into how clock dysfunction affects allergic disease. Allergic disease is also well-characterized by marked day-night changes in the clinical symptoms, laboratory parameters, and response to treatment. Readers who are interested in this subject are encouraged to refer to the reviews on how the circadian clock underpins a time of day–dependent variation in allergic reactions (14–16). The reviews highlight that oscillatory allergic reactions are generated by rhythmic expression of key molecules in the pathophysiology controlled by the circadian clock.

## Molecular Clocks in Mammalian Cells

In mammals, the circadian clock consists of several clock genes that are expressed in virtually all cell types, including cells in the skin, gut, airways, and immune system (11, 12, 17–19). At its core, the molecular clock consists of interlocking transcriptional–translational feedback loops (TTFLs) centered on the transcription factors BMAL1 and CLOCK (20, 21). BMAL1 heterodimerizes with CLOCK, and the heterodimer binds to E-box motifs (CANNTG) throughout the genome, driving the expression of thousands of genes, including *Period (Per1-3)* and *Cryptochrome (Cry1,2)*. The PER and CRY proteins form oligomers and move to the nucleus, where they inhibit BMAL1/CLOCK activity. This core loop, which takes ~24 h to complete, involves several post-transcriptional mechanisms such as enzymatic degradation of PER and CRY, and acts as a molecular oscillator to represent time of day within each cell.

Other than this core loop, a stabilizing loop within the clockwork regulating the timing and amplitude of BMAL1 is provided by the nuclear receptors RORα and REV-ERBα (Nr1d1) (20, 21). The BMAL1/CLOCK heterodimer activates transcription of RORα and REV-ERBα, which respectively activates and represses BMAL1 transcription.

Accordingly, the network of circadian proteins mediates periodic expression of thousands of genes (clock-controlled genes: CCGs) and regulates the timing of cellular activities on a 24-h cycle, ultimately dictating rhythmic physiology in various organs. The impact of the clockwork machinery is enormous: many CCGs, such as nuclear hormone receptors (NRs) like glucocorticoid receptor, are key regulators of major physiological processes (e.g., metabolism, immunity, development, reproduction) (22). Overall, 43% of all protein coding genes in mice shows circadian rhythms in transcription somewhere in the body, in an organ-specific manner (23).

## Coordination of the Multi-Oscillators

The human body contains ~40 trillion cells, each with a ~24-h clock. Thus, our body consists of a multi-oscillator network system. How are these astronomical numbers of clocks synchronized with other?

In mammals, the suprachiasmatic nucleus (SCN) of the hypothalamus (the central clock) serves as the master pacemaker in the body (20, 21, 24). The SCN receives afferent innervation about environmental light levels from the retina via the retinohypothalamic tract, which synchronizes the SCN clock via the cAMP response element binding (CREB) protein. In turn, the SCN transmits neuronal (e.g., sympathetic nerve activity) and hormonal (e.g., cortisol) signaling to synchronize peripheral clocks.

However, peripheral clocks can also be reset by non-SCN-derived hormones (e.g., insulin) and metabolic (e.g., NAD^+^ [nicotinamide adenine dinucleotide]^+^) signals connected with non-photic environmental cues such as food timing (25). For instance, livers of mice fed exclusively during the night or *ad libitum* (active phase) show a similar phase angle of cyclic liver gene expression, whereas feeding during the day almost entirely inverts the phase of liver oscillatory gene expression (26).

## How Does Circadian Disruption Occur?

As stated, circadian clocks become synchronized to a 24-h periodic environmental cue, which is called the zeitgeber (“time-giver” in German). Light and meal timing are strong zeitgebers. Accordingly, circadian rhythms in behavior, physiology, and metabolism become robust when the rhythmicity of internal clocks is coupled to that of external zeitgebers (20, 21, 24, 25). In other words, rhythms in the circadian system dampen when internal clock timing becomes mismatched with environmental zeitgebers.

Misalignment between the endogenous circadian clock and environmental cycles (or zeitgebers) compromises human mental and physical health (27). For instance, chronic circadian misalignment via night shift work, jet lag, or exposure to artificial light at night can precipitate or exacerbate mood disorders in susceptible individuals (28). Notably, night shift workers, who are exposed to aberrant light/dark conditions, irregular eating habits, and sleep disruption, are at a higher risk of cancer, cardiovascular and metabolic diseases, as well as sleep/psychiatric disorders (29). Thus, dramatic changes in modern lifestyles, including night shift work, nocturnal feeding, and shortness or irregularity of sleep impair our health via chronic circadian misalignment.

## Circadian Regulation of the Biology of Allergic Disease

### Clock Control of Epithelial Barrier Function Related to Allergic Disease

The epithelium in the skin, intestine, and airways acts as a physical, chemical, and biological barrier against pathogens, chemical agents, and allergens. Epithelial barrier dysfunction is critical for the initiation of allergic disease in many organs (2, 3, 5). In brief, disruption of epithelial barrier function increases epithelial permeability that enables entry of allergens into the body and activates the allergic immune response. Genetic (e.g., filaggrin deficiency) and non-genetic factors (e.g., protease activity of allergens, chemical agents, and injury/itch) contribute to the barrier disruption associated with allergic disease (30).

Below, I briefly summarize some examples of clock control of epithelial barrier function related to allergic disease. I will not discuss clock control of commensal bacteria, an important barrier against pathogenic and non-pathogenic insults in the epithelium of the skin, intestine, and airways, because this subject has been adequately reviewed elsewhere (31–33).

#### Skin

Our largest organ, the skin, is continuously exposed to numerous environmental irritants, including allergens. The skin barrier consists of many physical, chemical, and biological components, including filaggrin, lipid (e.g., ceramide), skin pH, tight junctions (TJs), anti-microbial peptides, commensal bacteria, and water content, most of which exhibit circadian rhythms (3, 34). In atopic dermatitis (AD), skin barrier function is impaired by several mechanisms, including filaggrin deficiency, injury (itch), and type 2 cytokines (e.g., IL-4 and IL-13) altering TJ protein expression, thereby precipitating allergic sensitization and inflammation (34, 35).

A clear example of circadian control of skin barrier function is aquaporin-3 (AQP3), which regulates water content by facilitating water and glycerol entry into keratinocytes. AQP3 expression in the skin is temporally controlled by CLOCK in mice and humans (36). Accordingly, stratum corneum hydration exhibits a significant 24-h rhythm in wild-type mice that is absent in *Clock-*mutated mice. Interestingly, *Clock*-mutated mice exhibit persistently reduced stratum corneum hydration (36). These findings illustrate that clock disruption can deregulate the components of skin barrier function.

#### Intestine

Intestinal epithelial cells function as the first line of defense against pathogenic and non-pathogenic microorganisms in the gut lumen (37). These epithelial barriers include TJs, mucus, and anti-microbial peptides. Several reports in humans and mice suggest that food allergy is associated with intestinal barrier dysfunction, which increases intestinal permeability to food compounds (38).

Several studies report that intestinal barrier function is under the circadian control. The epithelial TJ is a multiprotein complex that forms a selectively permeable seal between adjacent epithelial cells and limits paracellular passage of macromolecules, including allergens (39). CLOCK regulates expression of the TJ molecules Occludin and Claudin-1 in the mouse colon and controls circadian periodicity of intestinal permeability (40). In addition, in a mouse model of ovalbumin (OVA)-induced food allergy, OVA-induced allergic diarrhea exhibits daily variations associated with circadian periodicity in intestinal permeability (41), which implicates that the timing of food antigen intake can affect the severity of food allergy symptoms via clock control of intestinal permeability.

Importantly, *Clock*-mutated mice express lower levels of Occludin and Claudin-1 in the colon and are more sensitive than wild-type mice to the colonic injury induced by dextran sodium sulfate (DSS) (40). In addition, in a mouse model of chronic alcohol feeding, circadian disruption through genetics (*Clock* mutation) or environmental disruption (weekly 12-h phase-shifting) results in gut leakiness and exacerbates alcohol-induced gut leakiness and liver pathology (42). These findings illustrate that clock disruption can impair intestinal barrier integrity.

#### Airways

The airways are continuously exposed to physical, chemical, and biological insults in the air, including pathogens, pollutants, and allergens, and protect the host against them. Like the skin and intestine, the airway epithelial barrier consists of many physical, chemical, and biological components, including TJs, mucus-producing goblet cells, ciliated cells, and epithelial-derived cytokines/chemokines. Defects in the epithelial barrier are associated with asthma: for instance, bronchial biopsies from patients with asthma exhibit patchy disruption of TJs (43). Furthermore, these patients are more susceptible than healthy people to cigarette smoke or viral infection, which may precipitate asthma (44).

Pulmonary epithelial cells release cytokines and chemokines upon exposure to bacteria and viruses as a part of an innate defense response. The LPS-induced lung inflammatory response exhibits circadian periodicity in mice, relying on Bmal1 in bronchial epithelial cells (45). Since the epithelial response to LPS in the lung determines the development and severity of asthma (46, 47), Bmal1 regulation of the epithelial response to LPS may potentially explain the circadian nature of this disease.

Importantly, *Bmal1* deletion in bronchial epithelial cells enhances the LPS response in the lung without time-of-day oscillations (45). In addition, a loss-of-function mutation of REV-ERBα, a downstream target of Bmal1, in bronchial epithelial cells augments the LPS-induced inflammation in the lung, suggesting REV-ERBα as a key molecule that couples the core clock gene Bmal1 to innate immunity in the lung (48). Furthermore, responses to influenza infection are disrupted in mice with selective *Bmal1* deletion in bronchial epithelial cells (49). Thus, clock disruption can deregulate airway defense (barrier) mechanisms.

### Clock Control of Innate Immune Responses Related to Allergic Disease

#### Mast Cells/Basophil Response

Mast cells and basophils are innate immune cells that share many features, although they constitute a distinct lineage having several different roles in immune response and tissue homeostasis. In IgE-mediated allergic diseases such as allergic rhinitis, asthma, and food allergy, mast cells and basophils are the main effector cells and are activated by an IgE-dependent mechanism. IgE produced in response to allergen binds to the high-affinity IgE receptor (FcεRI) on the cell surface of mast cells and basophils, which triggers degranulation and production of cytokines/chemokines and lipid mediators, thereby shaping allergic inflammation (50).

The circadian nature of IgE-mediated allergic diseases is well-documented: in allergic rhinitis and asthma, symptoms, nasal or bronchial reactivity, and inflammatory activity become more pronounced in the early morning and at midnight (51). Skin hypersensitivity to allergens also varies with the time of day (52). These findings suggest that IgE-mediated allergic disease is under circadian control.

Consistent with the clinical observations, CLOCK controls the expression of FcεRI in mouse mast cells and releases mediators in a circadian manner upon IgE stimulation (53). Human basophils isolated from patients with asthma or allergic rhinitis exhibit a time-of-day–dependent variation in IgE-mediated responses (54, 55), although these findings may be controversial (56). *In vivo*, the extent of the passive cutaneous anaphylactic (PCA) reaction, a classical rodent model of IgE/mast cell-mediated allergic reaction, exhibits circadian variations (53, 57, 58).

Interestingly, the extent of the PCA reaction in *Per2*-mutated mice, mast cell–selective *Clock*-mutated mice, or mice fed only in the resting phase persistently exhibits a peak level equivalent to that of wild-type mice, but without circadian periodicity (53, 57–59). Thus, circadian disruption due to genetic alteration or irregular eating habits increases the susceptibility of mast cells to IgE throughout the day.

IL-33 activates mast cells and basophils via its receptor ST2. The crucial roles of the IL-33/ST2 axis in both IgE- and non-IgE–mediated allergic disease have been appreciated (60). CLOCK temporally gates the mast cell and basophil response to IL-33 via regulation of ST2 expression, which may also underlie circadian nature of allergic disease (61). Importantly, *Clock* mutation persistently enhances their response to IL-33 (61). Thus, clock disruption may enhance IgE- or IL-33-mediated mast cell/basophil responses.

#### Eosinophil Response

Eosinophils are main effector cells for the control of parasitic infections, but increasing evidence suggests that they play regulatory roles in tissue homeostasis/repairs and adaptive immune response. In allergic disease, eosinophils mediate their effector functions through several mechanisms: degranulation, extracellular traps, and cytolysis (62). Eosinophils require IL-5 survival signals, and anti-eosinophil monoclonal antibodies targeting IL-5 or IL5 receptor (IL5R) have been approved for clinical use against eosinophilic asthma (63).

Human eosinophils exhibit circadian oscillations in basal ECP (eosinophil cationic protein) expression and IL-8 (CXCL8) and CCL2 (The chemokine [C-C motif] ligand 2) release upon fMLP (N-formyl-methionyl-leucyl-phenylalanine) stimulation (64, 65). Furthermore, the number of blood and sputum eosinophils or serum IL-5 exhibits a time-of-day–dependent variation in asthma and control subjects or in mice (66–68). Interestingly, circadian variation of blood eosinophils has been linked to neuroendocrine and metabolic cycling (68). These findings are consistent with circadian control of eosinophil homeostasis, activation, and migration under steady states and in allergic disease, although few studies have addressed the direct roles of the clock in eosinophil function.

#### Macrophage Response

Macrophages are versatile innate immune cells that have phenotypic diversity and function in many different aspects of physiology and disease. Generally, the roles of macrophages in allergic disease remain obscure, but, in allergic asthma, macrophages may promote inflammatory responses associated with lung injury, fibrosis, and goblet cells hyperplasia (69).

Circadian clock components regulate various functions in macrophages, including cytokine secretion upon LPS challenge (70–72). About 8% of genes expressed in peritoneal macrophages are rhythmically transcribed, including essential elements in LPS/TLR4 signaling (71).

Interestingly, mice with *Bmal1*-deficient myeloid cells have markedly elevated eosinophil accumulation and serum BALF IL-5 expression in a model of allergic asthma (73). *Bmal1*-deficient macrophages produce more asthma-associated CCL2 and CXCL10 upon LPS stimulation than wild-type macrophages. Further, targeting REV-ERBα in myeloid cells exhibits persistently high pulmonary neutrophilic inflammation upon LPS challenge without time-of-day-dependent variations (48). Thus, clock disruption may enhance asthma by altering responses to LPS in macrophages, as well as in bronchial epithelial cells.

#### Innate Lymphoid Cell Response

Innate lymphoid cells (ILCs) are the most recently discovered family of innate immune cells that are ubiquitously distributed and are enriched in mucosal surface. ILCs consist of three different groups: group1, group 2, and group 3 ILCs (ILC1s, ILC2s, ILC3s), based on transcription factors required for the development, cytokine expression, and distinct effector functions.

ILC2s are key regulators of allergic immune responses (74). The epithelium-derived cytokines IL-25, IL-33, and TSLP activate ILC2s. The activated ILC2s release IL-5 and IL-13, which initiate and amplify allergic inflammation by activating eosinophils and epithelial cells. ILC2s in blood are more abundant in patients with asthma than in healthy subjects (75). The roles of clock in ILC2s remain unclear, but it is highly likely that ILC2s development and function are under circadian control, since RORα, a component of circadian clock, is involved in ILC2 differentiation (76) and IL-5 production from intestinal ILC2s exhibits circadian rhythms associated with blood eosinophil counts (68).

In contrast to ILC2s, several studies suggest that ILC3s, which provide mucosal defense through IL-22 and IL-17, are under strong control of the circadian clock. Bmal1 deficiency in ILC3s or disruption of light–dark cycles and feeding rhythms can deregulate gut ILC3 homeostasis, impairs epithelial reactivity, and deregulates the microbiome (77, 78). Because ILC3s have been implicated in obesity-induced asthma (79), clock disruption through metabolic disturbances in obese patients might enhance ILC3 responses and predispose patients to asthma.

#### Dendritic Cell Response

Dendritic cells (DCs) are the most potent professional antigen-presenting cells (APCs) *in vivo*, which can induce the activation and differentiation of naive T cells or induce immune tolerance. The ability of DCs to trigger immunity or tolerance by their co-stimulatory/inhibitory molecules is likely involved in the development of allergic disease. For instance, DC-expressing semaphorin 4, one member of the large family of secreted and membrane-bound glycoproteins that were initially implicated in axon guidance and neural development, drives Th2 response and are highly expressed in human asthmatic lung tissue (80).

The roles of clock in DCs remain largely unclear. One study shows that deletion of *Bmal1* in dendritic cells enhances the gut helminth *Trichuris muris* egg-specific protective Th2 response while suppressing the Th1 response in mice, suggesting that circadian machinery in DCs may contribute to Th1/Th2 balance (81). Thus, disruption of the DC-clock may affect Th1/Th2 balance, thereby having impact on allergic disease.

### Clock Control of Adaptive Immune Responses Related to Allergic Diseases

Lymphocytes (T cells and B cells) express clonally distributed receptors specific for diverse antigens (allergens) and are the key mediators of immune response. Among T cells, CD4^+^ T cells are called helper T cells (Th cells) because they help B cells or phagocytes to produce antibodies or destroy ingested microbes, respectively. Further, CD4^+^ T cells exhibit functionally distinct subsets called Th1, Th2, and Th17 that produce different cytokines and eliminate different types of pathogens.

In allergic disease, epithelial barrier disruption leads to production of innate cytokines such as IL-33 that skew DC phenotypes and activate innate immune cells (e.g., ILC2s, mast cells, and basophils), thereby eventually promoting the development of Th2 cell that secrete IL-4, IL-5, and IL-13. These cytokines promote B-cell isotype switching and IgE production, eosinophil accumulation, and mucus production from epithelial cells and shapes allergic inflammation (2).

Clock genes are rhythmically expressed in both T and B cells (82, 83) and temporally control lymphocyte trafficking and development, and immune responses against diverse pathogens (10–13). However, there have been limited studies addressing the roles of clock in T and B cell responses associated with allergic disease.

#### T Cell-Response

A mouse model of contact hypersensitivity in the skin depends on Th2 cells. In this model, *Clock*-mutant mice exhibit severe inflammation and increases in IL-4/IL-13 expression, mast cell number, and serum IgE levels relative to wild-type mice (84). As stated earlier, deletion of *Bmal1* in dendritic cells enhances the gut helminth *Trichuris muris* egg-specific protective Th2 response while suppressing the Th1 response. These findings suggest that clock proteins inhibit Th2 development and function, and that clock disruption may promote the Th2 response.

Th17 cells protect against bacterial and fungal infections at mucosal surfaces. In contrast to Th2 cells, circadian proteins clearly regulate Th17 cell differentiation (85). Deficiency of RORα or RORγ impairs Th17 development in mice. Consistent with this, mice maintained under chronic light cycle perturbations exhibit altered Th17 cell frequencies in the intestines relative to mice maintained under a normal light cycle. Since Th17 cells have been implicated in neutrophilic asthma (86), clock disruption may affect specific phenotypes of asthma.

Regulatory T cells (Tregs) contribute to prevention of allergic disease by regulating effector cells. In humans, genetic deficiency of Tregs shows allergic manifestations (87). Interestingly, mice subjected to perturbed light/dark cycles (6-h advance every 4 days) have fewer Tregs in the intestine than mice subjected to normal light/dark cycles; this is associated with the development of food allergy (88). Consistently, nurses with regular day/night-shift rotation exhibit an increased incidence of food allergy in comparison to nurses with no such rotation of work hours (88). Thus, clock disruption may decrease the number of Tregs, thereby precipitating allergic disease.

#### B-Cell Response

The connection between clock and B cell response is not well-studied. Bmal1-deficient mice have fewer pre-B cells in the bone marrow, as well as fewer B cells in the peripheral blood and spleen (89). This likely involves Bmal1 deficiency in the bone marrow microenvironment, but not B cell–intrinsic Bmal1 (89, 90). Thus, Bmal1 may play a role in the normal differentiation of B cells, but its relevance to allergy remains unknown.

**Figure 1:**
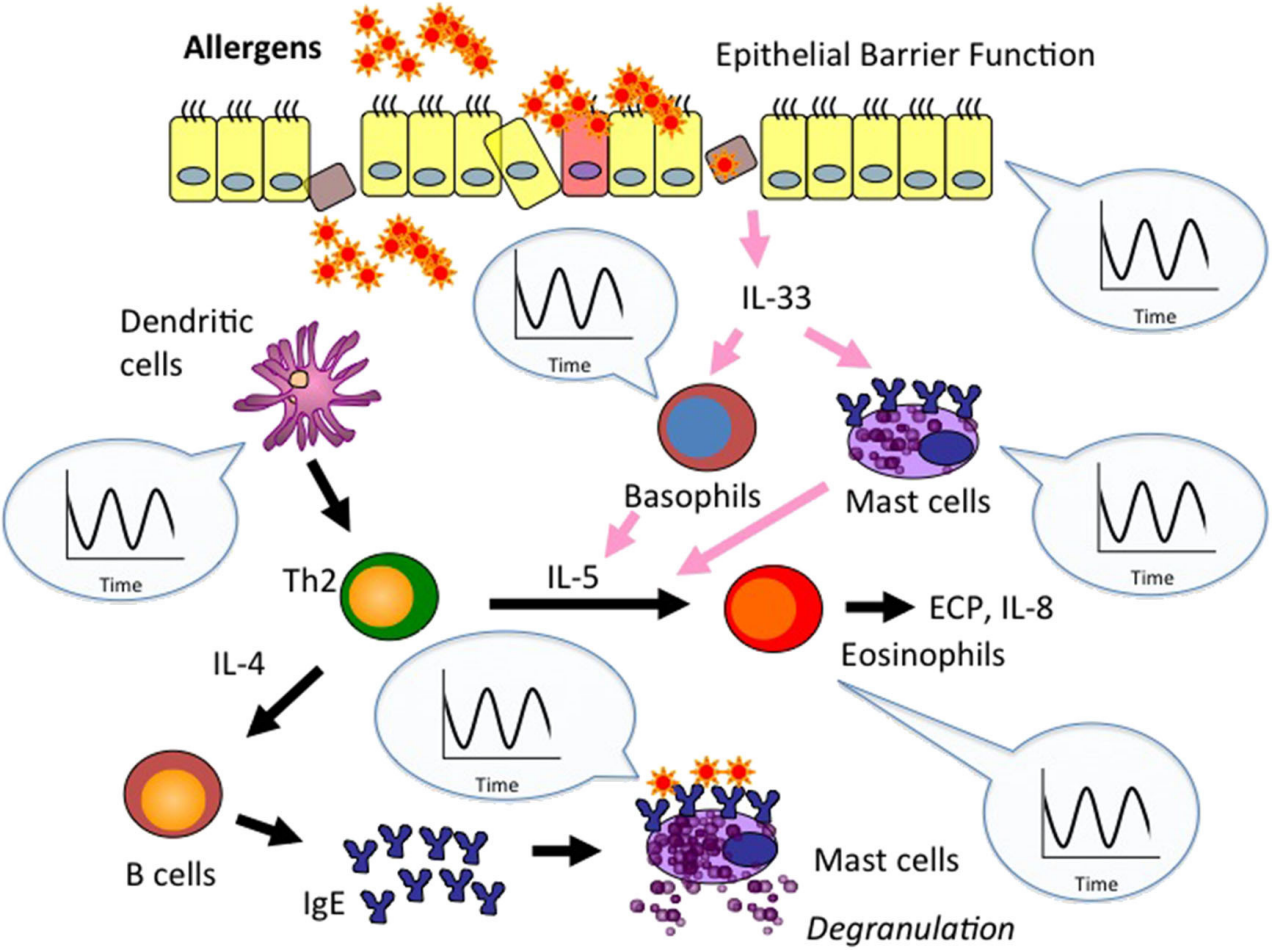
Circadian clock activity is embedded in epithelial barrier function and immune response associated with allergic disease. Epithelial barrier disruption enables entry of allergens into the body and leads to production of innate cytokines such as IL-33. The cytokines skew dendritic cell (DC) phenotypes, activate innate immune cells (e.g., mast cells, basophils), and promote Th2 development. Th2 cells secrete IL-4 and IL-5, which promote IgE production from B cells and eosinophil accumulation, respectively, and shape allergic inflammation. As stated in the text, circadian clock activity is embedded in the control of epithelial barrier function and immune cell responses associated with allergic disease. Circadian control of allergy-related epithelial barrier functions and innate immune cell responses (e.g., mast cells, basophils, eosinopihls, dendritic cells) has been extensively studied so far. Thus, we emphasize clock control of these elements in this figure. Please note that macrophage-, innate lymphoid cell-, T cell-, and B cell-responses are also controlled by the circadian clock. Accordingly, clock disruption may predispose allergic disease by deregulating epithelial barrier function and immune cell responses.

Collectively, these findings suggest that the circadian clock is strongly linked to two fundamental biological aspects of allergic disease, epithelial barrier function and immune responses (Figure 1).

## Allergic Immune Response May Temporally Complement Epithelial Barrier Function

Circadian gating of epithelial barrier function and immune response likely evolved to anticipate environmental physical, chemical, and biological insults (e.g., hot air, pollutants, pathogens) and to maximize host defense during the greatest time of the insults' exposure (11, 13). The temporal gating can also limit the costs of the defense response and may increase host fitness (11, 13).

**Figure 2:**
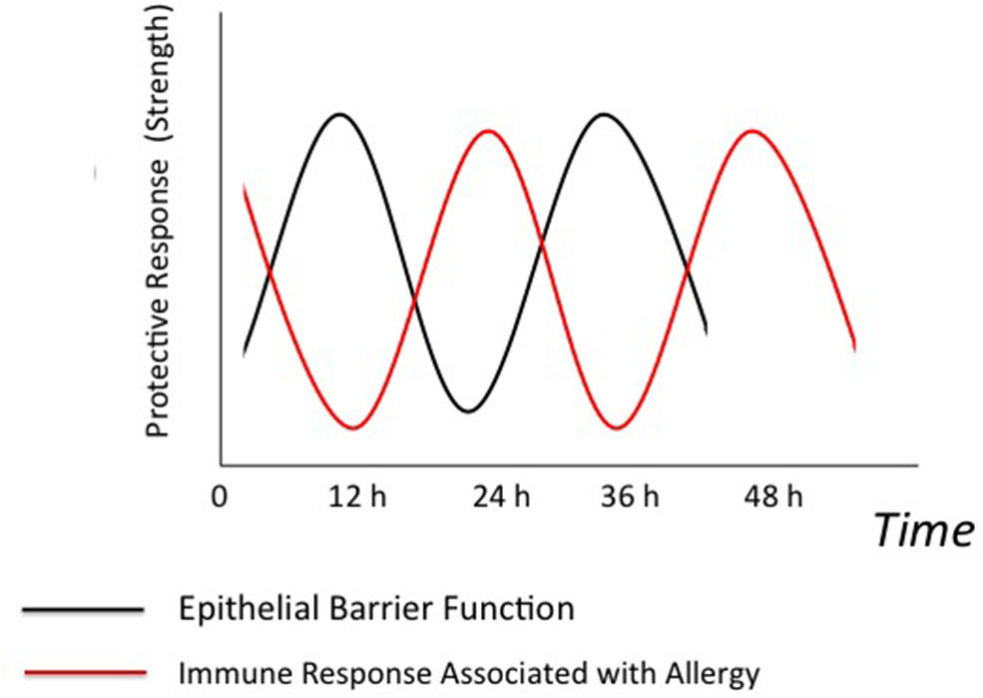
Possible temporal relationships between epithelial barrier function and allergic immune response. Epithelial barrier function and immune response associated with allergic disease (allergic immune response) are under control of the circadian clock. We speculate that epithelial barrier function and allergic immune response might temporally complement to each other. Briefly, clock system may maximize allergic immune response at the time-of-day when epithelial barrier function is weakest. This may be because the risk of attack by helminth and biting arthropods is highest at that time.

Allergic immune response, in particular IgE/mast cell-mediated response, is thought to evolve to confer protection against macroparasites such as helminth worms and biting arthropods such as mite and mosquito (91). Therefore, it is possible that the circadian control of immune responses associated with allergic disease deal with helminth and biting arthropods that themselves behave in a circadian manner (92, 93). We speculate that allergic immune response may temporally complement epithelial barrier function (Figure 2). For instance, skin, intestinal, and possibly airway barrier functions largely weaken in the resting phase (34, 40). This may be due to less exposure to noxious stimuli such as hot air and food-borne bacteria and fungi associated with feeding behavior in the resting phase than in the active phase. On the other hand, clock system may maximize mast cell response to IgE during the resting phase (14, 15) in order to prepare for the risk of attack by helminth and biting arthropods at that time. In other words, helminth and biting arthropods might evolve to attack target animals at the time-of-day when the animals' epithelial barrier function is weakest.

## Concluding Remarks: Circadian-Disrupted Modern Lifestyles May Promote Allergic Disease

The periodicity of allergic disease impacts on diagnostic and preventive aspects as well as on treatment effects. Recent studies highlight that circadian clock underpins allergic reaction, which likely confers the periodicity of allergic disease (14–16). Importantly, it is becoming clear that the circadian clock is a potent regulator of allergic reaction with more than a simple time-keeping role (16). Of note, several studies suggest that the circadian clock is strongly linked to two fundamental biological aspects of allergic disease, epithelial barrier function and immune responses. Thus, clock disruption can precipitate and enhance disease by deregulating the epithelial barrier and immune functions.

This new knowledge highlights circadian disruption as a new precipitating factor of allergic disease in modern society, in which our sleep, work, and eating habits are out of sync with endogenous circadian rhythmicity. This may partly explain why allergic disease is so prevalent in developed countries. Accordingly, the relationship between circadian biology and allergy will become an important area of research to understand allergic diseases in the modern era and exploring new ways to prevent or treat these disorders. In this context, we propose that lifestyle or therapeutic interventions that align the endogenous circadian clock with environmental cycles should be a part of the efforts to prevent or treat allergic disease in 24/7 society.

## Author Contributions

The author confirms being the sole contributor of this work and has approved it for publication.

## Conflict of Interest

The author declares that the research was conducted in the absence of any commercial or financial relationships that could be construed as a potential conflict of interest.
